# Novel *SETBP1* D874V adjacent to the degron causes canonical schinzel–giedion syndrome: a case report and review of the literature

**DOI:** 10.1186/s12887-024-04779-y

**Published:** 2024-05-06

**Authors:** Jing Zheng, Meiqun Gu, Shasha Xiao, Chongzhen Li, Hongying Mi, Xiaoyan Xu

**Affiliations:** 1 Department of Pediatrics, The First People’s Hospital of Yunnan Province, 157 Jinbi Road, Xishan District, Kunming, 650032 Yunnan Province China; 2 The Affiliated Hospital of Kunming University of Science and Technology, Kunming, China

**Keywords:** Schinzel-Giedion syndrome, *SETBP1*, Neonate, Hydronephrosis

## Abstract

Schinzel-Giedion syndrome (SGS) is a severe multisystem disorder characterized by distinctive facial features, profound intellectual disability, refractory epilepsy, cortical visual impairment, hearing loss, and various congenital anomalies. SGS is attributed to gain-of-function (GoF) variants in the *SETBP1* gene, with reported variants causing canonical SGS located within a 12 bp hotspot region encoding *SETBP1* residues aa868-871 (degron). Here, we describe a case of typical SGS caused by a novel heterozygous missense variant, D874V, adjacent to the degron. The female patient was diagnosed in the neonatal period and presented with characteristic facial phenotype (midface retraction, prominent forehead, and low-set ears), bilateral symmetrical talipes equinovarus, overlapping toes, and severe bilateral hydronephrosis accompanied by congenital heart disease, consistent with canonical SGS. This is the first report of a typical SGS caused by a, *SETBP1* non-degron missense variant. This case expands the genetic spectrum of SGS and provides new insights into genotype-phenotype correlations.

## Introduction

Schinzel-Giedion syndrome (SGS, OMIM 269,150) is a rare autosomal dominant genetic disorder first reported in 1978 [1]. Clinical features of SGS include distinctive facial appearance (commonly midface retraction), profound intellectual disability, refractory epilepsy, cortical visual impairment, hearing loss, and various congenital anomalies such as congenital heart disease, hydronephrosis, delayed neurological development, and skeletal dysplasia [2]. Liu et al. proposed revised diagnostic criteria for SGS, classifying it into three types based on clinical presentation and/or pathogenic *SETBP1* variant, enabling definitive diagnosis for patients with atypical clinical phenotypes via genetic testing and broadening the phenotypic spectrum [3].

Hoischen et al. confirmed that SGS is caused by variant in the *SETBP1* gene. In contrast to loss-of-function (LoF) variant leading to *SETBP1* haploinsufficiency disease (SETBP1-HD), characterized by hypotonia and mild motor developmental delay/intellectual disability (MIM: #616,078), known *SETBP1* variants causing SGS are gain-of-function (GoF) and located within a 12 bp hotspot region encoding *SETBP1* amino acid residues 868–871, which are associated with the canonical SGS phenotype. The *SETBP1* aa868-871 region functions as a degron, a signal regulating protein degradation, and pathogenic variants within the degron result in *SETBP1* protein accumulation. Missense variants of residues I871 and D868 have been demonstrated to be associated with the lowest and highest levels of pathogenic *SETBP1* protein, respectively, indicating a significant genotype-phenotype correlation in this region [4].

In this study, we report a patient with a typical SGS clinical presentation, who was found to carry a novel *de novo* heterozygous *SETBP1* D874V variant. The mutated residue is located near the degron region, but the patient presents with a classical clinical phenotype that could not be explained by the currently known genotype-phenotype correlations.

## Materials and methods

### Subjects

A female neonate who was admitted to the neonatal intensive care unit of First People’s Hospital of Yunnan Province was included in this study. She exhibited dysmorphic facial features (midface retraction, frontal bossing, and low-set ears), bilateral varus, syndactyly of the 4th and 5th toe, hydronephrosis, and congenital heart disease. We conducted follow-up for her clinical condition. The patient’s parents provided written informed consent to the study.

### Genetic tests

Two milliliters of the peripheral blood collected from the patient were used (anticoagulant: EDTA), and whole-exome sequencing was performed by Beijing Chigene Translational Medicine Research Center Co., Ltd (Beijing, China). Genomic DNA was extracted using the Blood Genome Column Medium Extraction Kit (Kangweishiji, China) according to the manufactural instructions. The extracted DNA samples were subjected to quality control using Qubit 2.0 fluorimeter and electrophoresis using 0.8% agarose gel for further protocol. Protein-coding exome enrichment was performed using xGen® Exome Research Panel v1.0 (IDT, Iowa, USA) that consists of 429,826 individually synthesized and quality-controlled probes, which targets 39 Mb protein-coding region (19,396 genes) of the human genome and covers 51 Mb of end-to-end tiled probe space. After target enrichment, high-throughput sequencing was performed on Illumina NovaSeq 6000 series sequencer (PE150), which was used to perform paired-end 150 bp sequencing, with a mean sequencing depth of 100X and sequencing coverage of 99%. Raw data were processed by the fastp software for adapters removing and low-quality reads filtering [5]. The paired-end reads were aligned to the Ensemble GRCh37/hg19 reference genome using the Burrows-Wheeler Aligner (BWA) software pacakage, and the GATK software was used reads calling. Detected single nucleotide polymorphisms (SNPs) and insertions and deletions (indels) not longer than 50 bp were then annotated using the ANNOVAR software. The common variants, with minor allele frequency (MAF) > 0.05, found in the 1000 Genomes Project and the ExAC and gnomAD databases were filtered out.

The primers designed for polymerase chain reaction (PCR) were as *SETBP1*-F, 5′-GGGAGCAGAAATCAAAAGAGTACC-3′ and *SETBP1*-R, 5′-CCAAAACCCAAAAGGGAATACACA-3′. Sanger sequencing was performed using the ABI 2720 DNA analyzer (USA). The NCBI BLAST algorithm was used for sequence alignment.

### Pathogenicity analysis of genetic variants

Computer software, including REVEL, SIFT, Polyphen2-HVAR, Polyphen2-HDIV, PROVEAN, and MutationTaster, were used to predict the deleterious effects of each variant on the protein function. Exomiser and Phenolyzer software were used to perform genotype-phenotype analysis. Homology modeling was performed using the Modeller software (https://salilab.org/modeller/) to analyze changes in the three-dimensional structure, and evolutionarily conserved regions were analyzed using UGENE software (http://ugene.unipro.ru/). Finally, the pathogenicity assessment and genetic interpretation of candidate gene variants were performed according to the American College of Medical Genetics and Genomics guidelines and criteria [5] for variant classification.

### Review of the literature

Variants and clinical features of previously reported cases with genetically diagnosed with SGS were collected. Data on these patients were retrieved from PubMed (http://www.ncbi.nlm.nih.gov/pubmed) using the search terms “Schinzel–Giedion syndrome” or “SGS” and “*SETBP1*” Only articles in English were included.

## Results

### Case presentation

**Fig. 1:**
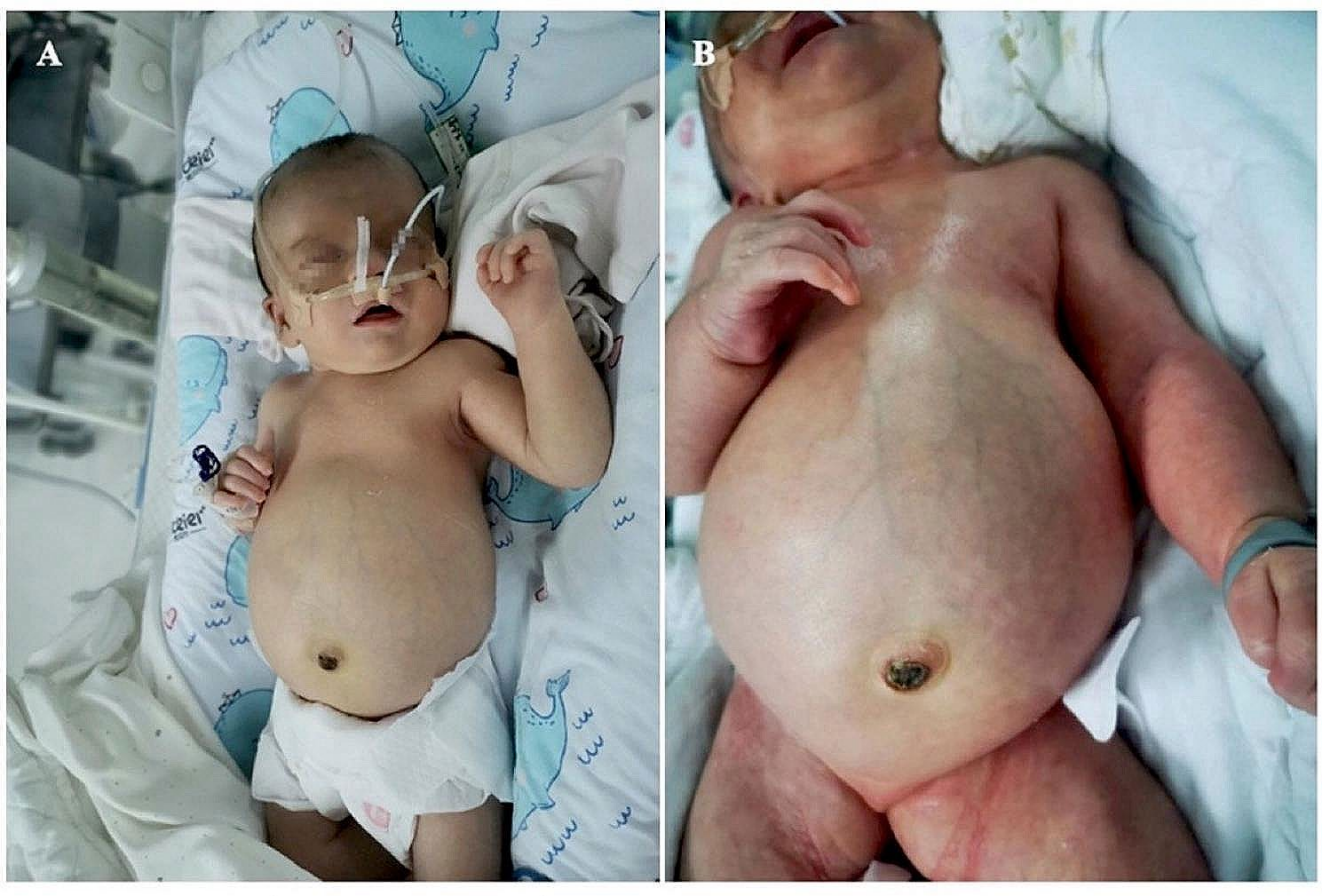
Phenotypic variants of the patient. (**A**) Characteristic midface retraction, frontal bossing, and low-set ears at 1 day after birth; (**B**) A grossly distended abdomen

**Fig. 2:**
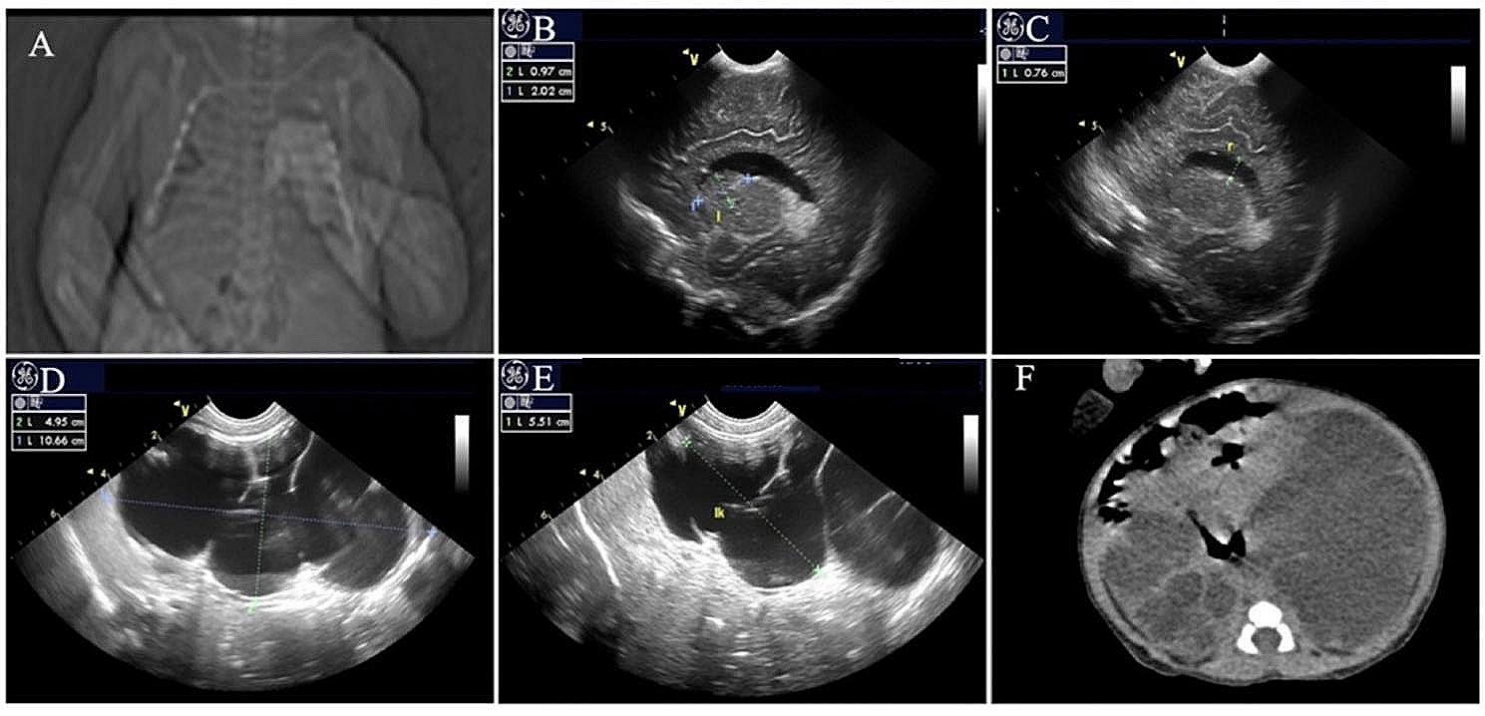
Photographs show the clinical features of the patient in this study. (**A**) Chest radiographs at 1 day after birth show wide ribsn (**B, C**) Color doppler ultrasonography at 10 days after birth shows widened bodies and anterior horns of lateral ventricles; (**D, E**) Color doppler ultrasonography at 10 days after birth shows severe hydronephrosis was found in both kidneys, bilateral pelvis and calyces were severely dilated with a “palette” appearancete (**F**) Abdominal CT at 1 day after birth shows Significant increase in the size of both kidneys, signs of severe hydronephrosis in both kidneys

The patient was admitted to the hospital with " high-risk delivery”, G1P1, born by cesarean section at a gestational age of 40^+ 4^ weeks, with a birth weight of 3430 g and an Apgar score of 9-9-9. Her parents were healthy, not consanguineous, and had no family history of specific diseases. The examination during pregnancy revealed that the mother suffered form gallbladder polyps and hypothyroidism.The fetus displayed severe hydronephrosis, pericardial effusion, and cauda equina cysts. Her physical examination upon admission showed stable vital signs, and stable breathing. Unique facial features included midface retraction, frontal bossing, and low-set ears (Fig. 1A). The anterior fontanelle was flat and soft, the size is 3.0 cm*3.0 cm. There wasn’t flaring of nares, nor perioral and fingertip cyanosis. The pulmonary examination showed no abnormalities, heart rate was 125 beats/min, heart rhythm was regular, and class II/6 systolic murmur could be heard in the precordial region. The abdomen was significantly enlarged (Fig. 1B), with an abdominal circumference of 38 cm, 2–3 bowel sounds/min, increased muscle tone of the limbs, limited abduction of both upper and lower limbs, bilateral varus, syndactyly of the 4th and 5th toe and vulvar malformation (dysplasia of the labia majora). During hospitalization, the patient had normal lab results for full biochemistry profile, routine blood, urine, and stool examinations, and thyroid function tests. In addition, tandem mass spectrometry screening of blood and urine samples showed normal results. Chest radiographs revealed broad bones (Fig. 2A). Color Doppler echocardiography showed atrial septal defect, patent ductus arteriosus, right atrial and right ventricular enlargement. Moreover, color Doppler ultrasonography of the urinary system detected bilateral severe hydronephrosis (Fig. 2 D, E). Abdominal CT showed a significant increase in the size of both kidneys and signs of severe hydronephrosis in both kidneys (Fig. 2F). Cranial color Doppler ultrasonography showed grade II-IVH on the left side and moderate enlargement of bilateral lateral cerebral ventricles (Fig. 2B, C). Video electroencephalogram (EEG) showed normal results. No ocular fundus abnormalities were observed. Moreover, both ears did not respond to the rapid auditory brainstem response test. Due to the deformities of the heart, kidney, bone and other organs of the child, congenital genetic metabolic diseases could not be excluded. After obtaining the consent of the family members, the Whole-Exome Sequencing was improved. The child was treated with nasal catheter oxygen and anti-infection during the hospitalization. The child had difficulty in early feeding(weak sucking reflex) and could be fully fed orally before discharge, but the sucking power was also poor. Her parents abandoned treatment and was discharged from the hospital 13 days after birth. 8 months after birth, in the outpatient follow-up, the child presented with delayed gross motor development and no seizures, and the patient’s family members refused to perform a cranial MRI.

### Result of genetic tests

Whole-exome sequencing suggested a heterozygous variant (c.2621 A > T, p.Asp874Val) in exon 4 of *SETBP1* (NM_015559). This variant was a missense variant. The detected variant was not found in many databases including the 1,000 Genomes Project, ExAC gnomAD and dbSNP databases.

### Analysis of the pathogenicity of gene variant

**Fig. 3:**
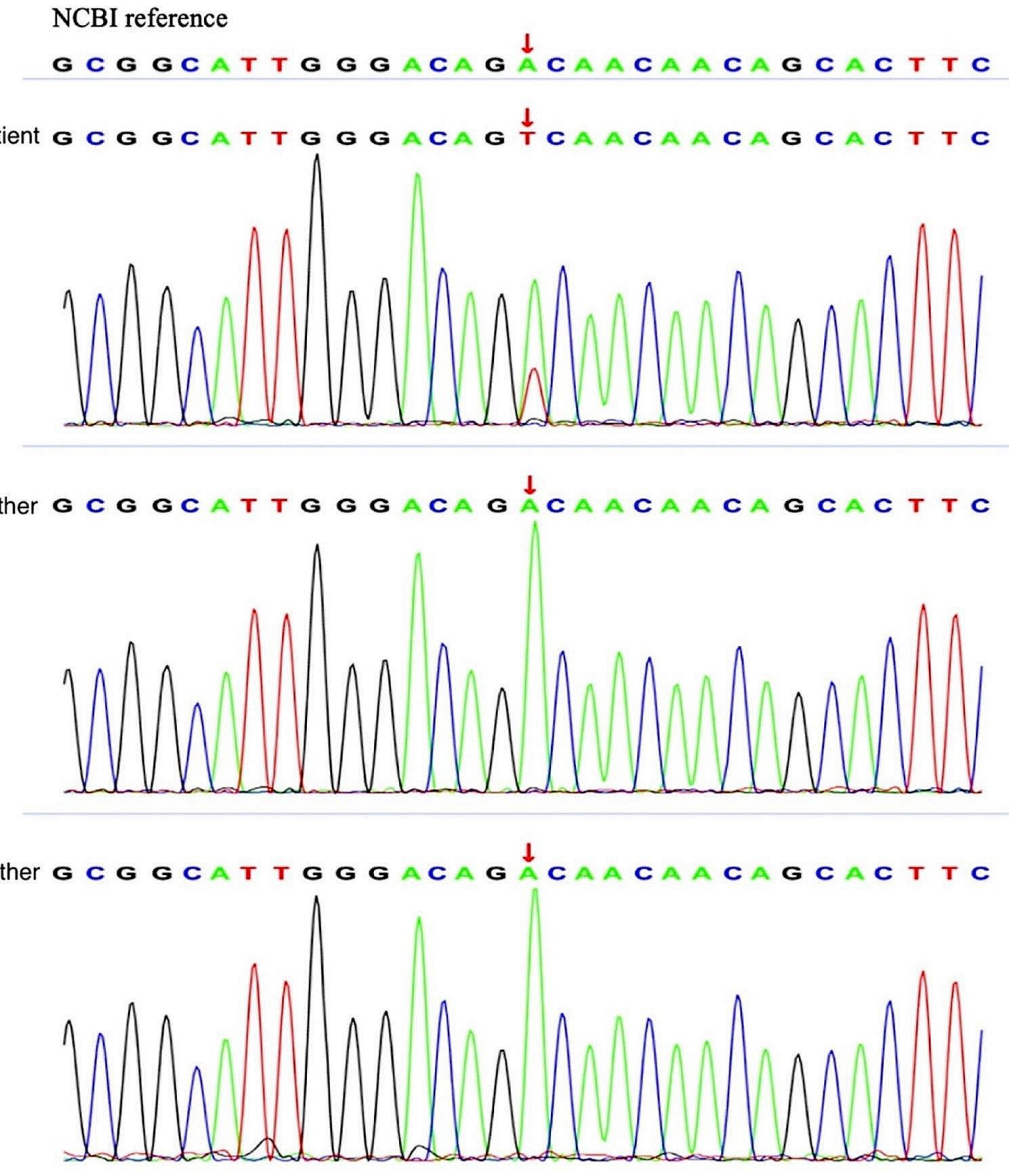
Sanger sequencing confirms de novo c.2621 A > T variant(p.Asp874Val) in the *SETBP1* gene in the patient

Sanger sequencing of the genome of the patient and her parents suggested that the patient had a de novo variant; As both parents had the wild-type gene (Fig. 3). Some silico predictions, including REVEL, SIFT, Polyphen2-HVAR, Polyphen2-HDIV, PROVEAN, and MutationTaster, suggested the deleterious effects of this variant on protein function. There has been no previous report of such variants. This variant was classified as likely pathogenic according to American College of Medical Genetics and Genomics guidelines (the supporting evidence for likely pathogenicity was PS2 + PM1 + PM2_Supporting + PP3).

**Fig. 4:**
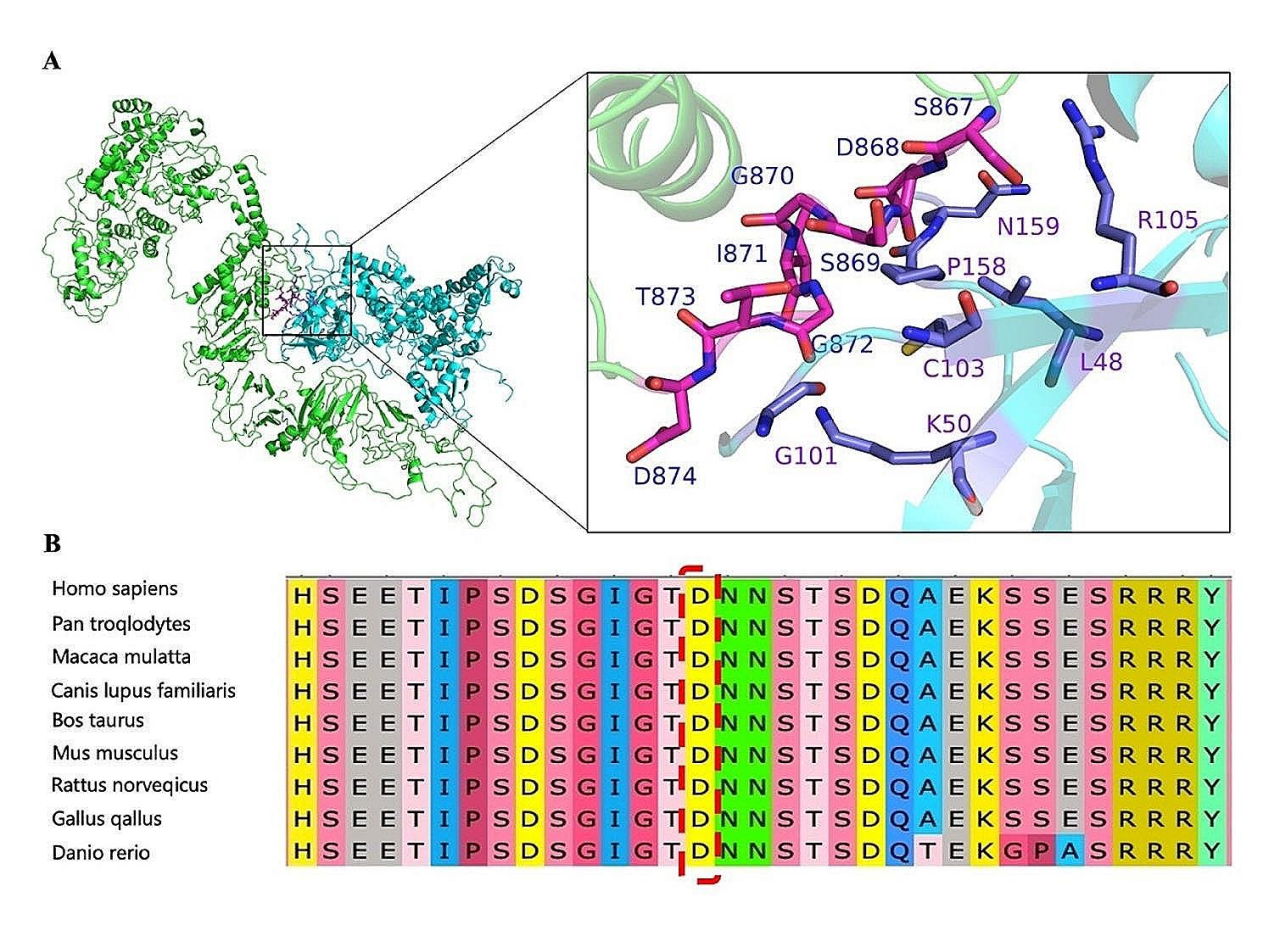
In-silico analysis of *SETBP1* c.2621 A > T/p.Asp874Val variant. (**A**) Protein-DNA/RNA docking using HDOCK server (http://hdock.phys.hust.edu.cn/) (hybrid algorithm of template-based modeling and ab initio free docking). As shown in Fig. 4A: Green represents *SETBP1* protein and sky blue represents E3 ubiquitin ligase. S876, D868, S869, G870, I871, G872, T873 and D874 of *SETBP1* form interactions with L48, K50, G101, C103, R105, P158 and N159 of E3 ubiquitin ligase, which in turn promote *SETBP1* binding to E3 ubiquitin ligase and thus ubiquitination occurs. The hotspot variants D868, S869, G870 and I871 of *SETBP1* gene significantly affect the binding of *SETBP1* to E3 ubiquitin ligase, thereby affecting ubiquitination. The D874V variant reported in this paper is also in the vicinity of the *SETBP1* gene binding E3 ubiquitin ligase, which may also affect the binding of *SETBP1* protein to E3 ubiquitin ligase and thus affect *SETBP1* protein ubiquitination, ultimately leading to the development of the disease. (**B**) Alignment of the *SETBP1* sequences revealed that the amino acid residues at position 874 were strictly conserved

Protein-DNA/RNA docking using HDOCK server(http://hdock.phys.hust.edu.cn/) (hybrid algorithm of template-based modeling and ab initio free docking) is shown in Fig. 4A. Green represents *SETBP1* protein and sky blue represents E3 ubiquitin ligase, which promotes ubiquitination and degradation of *SETBP1* protein. When mutated, *SETBP1* protein fails to bind to E3 ubiquitin ligase, leading to protein overexpression. As shown in Fig. 4A: S876, D868, S869, G870, I871, G872, T873 and D874 of *SETBP1* form interactions with L48, K50, G101, C103, R105, P158 and N159 of E3 ubiquitin ligase, which in turn promote *SETBP1* binding to E3 ubiquitin ligase and thus ubiquitination occurs. The hotspot variants D868, S869, G870 and I871 of the *SETBP1* gene significantly affect the binding of *SETBP1* to E3 ubiquitin ligase, thereby affecting ubiquitination. The D874V variant(p.Asp874Val) reported in this paper is also in the vicinity of the *SETBP1* gene binding E3 ubiquitin ligase, which may also affect the binding of *SETBP1* protein to E3 ubiquitin ligase and thus affect *SETBP1* protein ubiquitination, ultimately leading to the development of the disease.Alignment of the *SETBP1* sequences revealed that the amino acid residues at position 874 were strictly conserved (Fig. 4B).

The aforementioned data, the patient’s clinical manifestations, and the *SETBP1* variant status indicated that SGS was caused by a heterozygous variant (c.2621 A > T, p.Asp874Val) in *SETBP1*.

### Review of the literature

We evaluated a total of 20 articles and 60 patients. All data were curated in Table 1.

**Table 1:** Clinical features and identified *SETBP1* variants in previously reported and present studies of SGS cases

cDNA	Protein	Case	Clinical manifestations					Whether the clinical phenotype is consistent with classical SGS	References
			Facial dysmorphisms	Neurological abnormalities		Urological findings	Characteristic skeletal findings		
				Developmental delay	Seizures				
**Hotspot variants (residues 868–871)**									
c.2602G > A	p. Aap868Asn	15	15/15(100%)	14/14(100%)	15/15(100%)	14/15(100%)	7/9(78%)	15/15(100%)	4, 6, 7, 8, 9
c.2602G > T	p. Aap868Thr	1	1/1 (100%)	1/1 (100%)	1/1 (100%)	1/1 (100%)	1/1 (100%)	1/1 (100%)	4
c.2603 A > C	p. Aap868Ala	1	1/1 (100%)	1/1 (100%)	1/1 (100%)	1/1 (100%)	1/1 (100%)	1/1 (100%)	6
c.2605 A > G	p. Ser869Gly	1	1/1 (100%)	1/1 (100%)	1/1 (100%)	1/1 (100%)	NA	1/1 (100%)	10
c.2605 A > T	p. Ser869Cys	1	1/1 (100%)	1/1 (100%)	1/1 (100%)	1/1 (100%)	NA	1/1 (100%)	11
c.2606G > A	p. Ser869Asn	1	1/1 (100%)	1/1 (100%)	1/1 (100%)	1/1 (100%)	1/1 (100%)	1/1 (100%)	4
c.2607 C > G	p. Ser869Arg	1	1/1 (100%)	1/1 (100%)	1/1 (100%)	1/1 (100%)	NA	1/1 (100%)	4
c.2608G > A	p. Gly870Ser	14	13/13(100%)	12/12(100%)	12/13(92%)	13/14(93%)	5/5 (100%)	14/14(100%)	4, 6, 12–16
c.2608G > T	p. Gly870Cys	1	1/1 (100%)	1/1 (100%)	1/1 (100%)	1/1 (100%)	0/1 (0%)	1/1 (100%)	17
c.2609G > A	p. Gly870Asp	3	3/3 (100%)	3/3 (100%)	3/3 (100%)	1/1 (100%)	2/2 (100%)	3/3 (100%)	4,6
c.2612T > C	p. Ile871Thr	15	14/14(100%)	10/11(91%)	13/14(93%)	13/15(87%)	7/8 (88%)	14/15(93%)	4,6,18,19,20
c.2612T > G	p. Ile871Ser	2	2/2 (100%)	2/3 (100%)	1/1(100%)	1/2 (50%)	NA	1/2(50%)	21,22
Total		56	54/54(100%)	48/49(98%)	52/54(96%)	51/56(91%)	24/28(86%)	54/56(96%)	
**Non-Hotspot variants**									
c.1181-1184insA	p.Glu394GlufsX	1	1/1(100%)	1/1(100%)	1/1(100%)	0/1(0%)	0/1(0%)	0/1(0%)	3
c.2572G > A	p. Glu858Lys	1	1/1(100%)	1/1(100%)	1/1(100%)	0/1(0%)	NA	0/1(0%)	23
c.2584G > A	p. Glu862Lys	1	1/1(100%)	1/1(100%)	0/1 (0%)	0/1(0%)	NA	0/1(0%)	4
c.2601 C > A	p. Ser867Arg	2	2/2(100%)	1/1(100%)	2/2(100%)	0/1(0%)	NA	0/1(0%)	4,9
c.2618 C > T	p. Thr873Ile	1	1/1(100%)	1/1(100%)	0/1 (0%)	0/1(0%)	NA	0/1(0%)	4
c.2621 A > T	p. Asp874Val	1	1/1(100%)	1/1(100%)	0/1 (0%)	1/1(100%)	1/1(100%)	1/1(100%)	**Our case**
Total		7	7/7(100%)	6/6(100%)	4/7(57%)	1/7(14%)	1/7(14%)	1/7(14%)	

## Discussions

Liu et al. have updated the diagnostic criteria for Schinzel-Giedion (SGS). The revised criteria now classify SGS into three distinct types based on clinical observations and the presence of the *SETBP1* variant [3]. SGS type I, also known as the classic type, is characterized by the hallmark clinical features, including developmental delays and distinctive facial morphology such as a prominent forehead, midface retraction, and low-set ears, as well as the presence of hydronephrosis or two of the four characteristic skeletal anomalies, including a sclerotic skull base, wide occipital synchondrosis, increased cortical density or thickness, and broad ribs. This classification aligns with the previously proposed diagnostic criteria by Lehman and colleagues. Type II refers to as an intermediate phenotype of SGS, is diagnosed in patients with development delays and the distinctive facial features, but without the presence of hydronephrosis or typical skeletal abnormalities, with the presence of the *SETBP1* variant. Type III, also known as the simple type, is diagnosed in patients with the *SETBP1* variant and developmental delays, with expressive language delay being the most prominent feature. These revised criteria will aid in the accurate diagnosis and management of SGS.

As far as we know, all reported cases of classical SGS meeting the diagnostic criteria proposed by Lehman and colleagues have exhibited missense variants within a 12-basepair hotspot located in exon 4 of the *SETBP1* gene (Table 1) [4, 6–23]. This hotspot, which encodes four amino acid residues (D868, S869, G870, and I871), known as the degron, is located within the SKI homologous region of the *SETBP1* protein and is a critical site for substrate recognition by the cognate SCF-β-TrCP E3 ubiquitin ligase [4, 12]. Variants within the degron prevent the binding of *SETBP1* protein to E3 ubiquitin ligase, leading to protein overexpression [24, 25]. Patients with missense variants in residues 862, 867, and 873 near the hotspot region exhibit a milder SGS phenotype, and the proximity of the mutated position to the degron is associated with clinical overlap with the classic SGS phenotype [4, 7]. Notably, D874V found in this study is adjacent to the degron, which is inconsistent with the previous knowledge.

To date, 63 cases of genetically confirmed SGS have been reported, including 16 missense variants and 1 insertion variant (Table 1). *SETBP1* D874V reported in this paper is a novel variant and the first report of *SETBP1* non-hotspot variant identified in a canonical SGS case. Being highly conserved in different species, D874 might have an important biological role. In addition, protein model analysis of the variant suggests that the hotspot variants D868, S869, G870 and I871 of the *SETBP1* gene significantly affect the binding of *SETBP1* to E3 ubiquitin ligase, thereby affecting ubiquitination. The D874V variant reported in this paper is located in proximity of the bliding site for E3 ubiquitin ligase, which may also affect the binding of *SETBP1* protein to E3 ubiquitin ligase and thus affect *SETBP1* protein ubiquitination, ultimately leading to the development of the disease.

Diagnosing SGS in the neonatal period can be challenging due to the presence of non-specific symptoms, including genital abnormalities, reduced sucking ability, decreased muscle tone, and EEG waveform abnormalities, in addition to the typical clinical manifestations of SGS [12]. In a 2022 case report by Yang et al. [10], a neonatal patient with midface retraction and developmental delay was diagnosed with “non-classical” SGS based on the absence of hydronephrosis and skeletal abnormalities at birth, and the presence of a *SETBP1* gene variant, S869G, according to the Lehman diagnostic criteria. However, after 18 months of follow-up, bilateral hydronephrosis was detected by color Doppler ultrasonography, leading to a revised diagnosis of classical SGS. The authors emphasize the importance of long-term follow-up to observe the evolution of phenotypes diagnosed by early molecular testing, as phenotypic changes are common, particularly in infants. Out of 56 patients with SGS exhibiting variants in the degron sequence (hotspot variants sequence), five did not have hydronephrosis. According to Yang et al.‘s case report, progressive hydronephrosis may occur in long-term survivors, albeit at a slower development rate. However, the patient reported in this study developed severe hydronephrosis in the neonatal period, underscoring the need for close monitoring of renal function during later follow-up.

The *SETBP1* protein is expressed throughout the body, but its levels are highest during brain development before birth, when nerve cells undergo proliferation and migration to specific regions of the brain. Variants in the *SETBP1* gene can result in severe neurological developmental abnormalities, given its critical role in this process. A study by Banfi F et al. revealed that SETBP1 variants lead to the accumulation of the SETBP1 and SET proteins and the consequent P53 inhibition in neural cells. These molecular changes promote the onset of cancer-like behavior in neural progenitors that accumulate widespread DNA damage without programmed cell death engagement [26]. Neurodevelopmental delay is a hallmark characteristic of SGS, with asphyxia, feeding difficulties, and recurrent apnea being common symptoms in the neonatal period [11]. A stud*y by* Wong MM et a*l* illustrated that the variants that carrying SETBP1 missense variants outside the degron, cause a clinically and functionally variable developmental syndrome, showing only partial overlaps with classical SGS and SETBP1-HD, and primarily characterised by intellectual disability, epilepsy, speech and motor impairment [27]. The incidence of developmental delay, epilepsy, and expressive language delay is extremely high with increasing age, with 97% (56/58) of reported cases of children with SGS presenting with neurological developmental abnormalities, including developmental delay and seizures. In this study, cranial color Doppler ultrasonography showed moderate enlargement of bilateral lateral cerebral ventricles and a normal EEG waveform during the neonatal period. However, the patient’s family members did not approve of performing a cranial MRI. Currently, at 8 months of age, the patient exhibits retardation of gross motor development but has not experienced epileptic seizures. Long-term follow-up visits are required to monitor the patient’s progress. These findings highlight the importance of early diagnosis and appropriate imaging studies in the management of SGS, particularly in patients with a suspected neurological developmental abnormality.

In summary, this study reports the first case of canonical SGS in a Chinese neonate caused by a novel *SETBP1* non-degron region variant, D874V. This finding expands the genetic spectrum of SGS and provides a new case for investigating genotype-phenotype correlations in SGS.

## Data Availability

The datasets presented in this article are not readily available because of privacy restrictions (the guardians of patients are reluctant to authorize the release of all the raw data of whole-exome sequencing, but agree to contribute positive sanger-sequencing results). Requests to access the datasets should be directed to the corresponding author.

## Abbreviations

SGS: Schinzel-Giedion syndrome
GoF: Gain-of-function
LoF: Loss-of-function
EEG: Electroencephalogram
